# Management of Ogilvie’s Syndrome: A Network Meta-Analysis

**DOI:** 10.3390/jcm15083177

**Published:** 2026-04-21

**Authors:** Orestis Ioannidis, Christos Chatzakis, Ioannis Mitrogiannis, Elissavet Anestiadou, Aliki Brenta, Savvas Symeonidis, Stefanos Bitsianis, Efstathios Kotidis, Manousos George Pramateftakis, Ioannis Mantzoros, Stamatios Angelopoulos

**Affiliations:** 1 Fourth Surgical Department, Aristotle University of Thessaloniki, 54124 Thessaloniki, Greecealikibrenta@gmail.com (A.B.);; 2 Obstetrics and Gynecology Department, Ioannina University, 45110 Ioanina, Greece

**Keywords:** Ogilvie’s syndrome, acute colonic pseudo-obstruction, neostigmine, colonoscopic decompression, network meta-analysis

## Abstract

**Background/Objectives:** Ogilvie’s syndrome, or acute colonic pseudo-obstruction (ACPO), is defined by acute colonic dilatation without mechanical obstruction and carries a risk of ischemia and perforation if not promptly managed. Treatment strategies include conservative supportive care, neostigmine administration, colonoscopic decompression, and combinations of these approaches. The aim of this study was to compare the relative effectiveness of these interventions for symptom resolution using a network meta-analysis. **Methods:** A systematic search of PubMed, Embase, Scopus, the Cochrane Central Register of Controlled Trials, ClinicalTrials.gov, and grey literature was conducted from inception in February 2025 to November 2025. Prospective cohort and case–control studies evaluating conservative supportive care, neostigmine, colonoscopic decompression, or their combinations were included. A random-effects network meta-analysis was performed using odds ratios (ORs) with 95% confidence intervals (CIs). Treatment ranking was assessed using surface under the cumulative ranking curve (SUCRA) values. Risk of bias was evaluated using the Newcastle–Ottawa Scale (NOS). **Results:** Four studies comprising 172 patients were included. Compared with supportive care alone, supportive care combined with neostigmine was associated with higher odds of symptom resolution (network OR 13.86, 95% CI 3.06–62.83). Supportive care combined with colonoscopic decompression demonstrated an even greater effect (network OR 65.65, 95% CI 11.70–368.50). Colonoscopic decompression versus neostigmine yielded a network OR of 4.74 (95% CI 1.17–19.25). SUCRA rankings indicated that colonoscopic decompression combined with supportive care had the highest probability of being the most effective strategy. **Conclusions:** Active interventions, particularly colonoscopic decompression or neostigmine combined with supportive care, were associated with a higher incidence of symptom resolution compared to supportive care alone. Larger comparative studies are needed to confirm these findings and refine treatment selection.

## 1. Introduction

Ogilvie’s syndrome, or acute colonic pseudo-obstruction (ACPO), is characterized by acute colonic dilatation in the absence of mechanical obstruction and primarily affects hospitalized, post-operative and elderly patients with an estimated incidence of approximately 100 cases per 100,000 hospital admissions [1,2]. The condition is linked with significant morbidity/mortality rates (8–15%) due to the risk of ischemia and perforation, particularly in cases of progressive colonic distension. The risk of colonic perforation is higher when cecal diameter exceeds 10–12 cm and when the distension has been present for greater than six days. The duration of dilation is more important than the absolute colon diameter. The later, in combination with prolonged hospitalization and increased healthcare resource utilization, highlights the need for timely diagnosis and essential management [3,4,5].

Management of Ogilvie’s syndrome typically follows a stepwise approach [6]. Initial treatment consists of conservative supportive care, including bowel rest, correction of electrolyte abnormalities, and decompression [4,5,7]. In patients who fail to respond, pharmacological therapy with neostigmine or colonoscopic decompression is commonly employed [7,8]. Although both interventions are widely used, evidence comparing their relative effectiveness, either alone or in combination, remains limited and heterogeneous.

Most existing studies assess individual treatment modalities or rely on narrative synthesis, restricting direct comparison across available strategies [8,9,10]. Scarce evidence is available in published literature mainly limited by small sample sizes, various study designs, and a lack of direct head-to-head comparisons. A network meta-analysis provides a more comprehensive, robust assessment of relative treatment effectiveness by giving the opportunity to simultaneously compare multiple interventions. This study aimed to compare conservative supportive care, neostigmine, colonoscopic decompression, and their combinations for symptom resolution in patients with Ogilvie’s syndrome using a network meta-analytic approach.

## 2. Materials and Methods

### 2.1. Reporting Guidelines and Registration

This meta-analysis adhered to the guidelines outlined in the PRISMA extension statement for network meta-analyses (Table S1) [11].

### 2.2. Inclusion and Exclusion Criteria

Inclusion Criteria: All clinical studies (cohort, case–control prospective studies) evaluating the effect of different management strategies for Ogilvie’s syndrome in adults (age ≥ 18 years) were eligible for inclusion. Endoscopic decompression, neostigmine administration, conservative supportive care, and/or a combination of these methods were the interventions of interest. Studies were not excluded based on their publication date and there were no language limitations.

Exclusion Criteria: Studies involving pediatric populations, case reports, small case series, reviews, editorials, and studies lacking clear outcome definitions or insufficient data on symptom resolution were excluded. Additionally, studies were excluded if they did not meet a minimum methodological quality threshold.

### 2.3. Primary and Secondary Outcome Measures

This study identified the resolution of symptoms as a primary outcome, defined as clinical improvement in abdominal distension accompanied by radiological evidence of colonic decompression and/or return of bowel function. Both positive and negative associations were considered, with no restrictions on language or publication date. There were no secondary outcomes.

### 2.4. Search Methods

A predefined search strategy was used to identify eligible studies in electronic databases published from inception to 17 November 2025 ([PubMed, Embase, Scopus, US Registry of clinical trials (www.clinicaltrials.gov), accessed on 15 March 2025], Cochrane Central Register of Controlled Trials (Central), and sources of grey literature, using combinations of the terms “Ogilvie syndrome”, “Acute colonic pseudo-obstruction”, “management”, “treatment”, “conservative management”, “neostigmine”, “endoscopy” and “colonoscopy”). An automated search using PubMed’s “search for related articles” feature to supplement the searches wad done. Title and abstract screening were conducted in order to identify potentially eligible articles for the next step of full-text evaluation. Moreover, our search was complemented with reference citation of the retrieved articles for the relevant literature. All studies were carefully compared to prevent the inclusion of duplicate/overlapping samples. In cases of overlap, the study with the larger number of cases was considered for inclusion. A detailed description of our search strategy is presented in Table 3.

### 2.5. Study Selection

Two reviewers (CC and IM) independently assessed the eligibility of the studies based on the aforementioned criteria. Discrepancies among the reviewers were solved by consultation of a third reviewer (OI) through arbitration.

### 2.6. Data Extraction

The following information was extracted during data extraction from each study: first author, year, sample size, age, gender, primary disease, type of surgery, study type, duration of treatment, intervention details, data from the control group, and outcomes. Two reviewers (IM and CC) independently assessed the quality of each study. A predefined data extraction form was used to evaluate the study characteristics of the primary studies. In case of disagreements, a consensus was reached through extensive discussion between the two reviewers.

### 2.7. Risk of Bias

The included observational studies were assessed for methodological quality using the Newcastle–Ottawa Scale (NOS), which evaluates the risk of bias across three broad domains: selection of study groups, comparability of groups, and ascertainment of exposure or outcomes, as appropriate [12]. The selection domain assessed the representativeness of the exposed cohort or cases, the selection of the non-exposed cohort or controls, and the ascertainment of exposure. Comparability focused on the extent to which studies controlled for potential confounding factors. The outcome or exposure (for cohort or case–control studies, respectively) domain evaluated the method of outcome assessment, adequacy of follow-up, and completeness of data. Studies were awarded a maximum of nine stars. Studies receiving 7–9 stars were considered to be at low risk of bias, those with 5–6 stars were considered to have a moderate risk of bias, and studies with fewer than 5 stars were considered to be at high risk of bias.

### 2.8. Geometry of the Networks

For the primary outcome, a network plot was created to include all groups that received endoscopic intervention, neostigmine administration, conservative supportive care or a combination of them. Each group was represented by nodes, and the comparisons between them were represented by edges. The size of a node was proportionate to the number of patients, while the thickness of the edges reflected the number of studies evaluating each intervention (endoscopic intervention, neostigmine administration, conservative supportive care or a combination of them). The network plots were generated using the “netgraph” command from the “netmeta” package in R (R: a language environment for statistical computing, R Foundation for Statistical Computing, Vienna, Austria).

### 2.9. Assessment of Transitivity

Transitivity, which consists of an important assumption in network meta-analysis, suggests that valid comparisons between two interventions can be made through indirect routes involving either one or more several intermediate comparators. Transitivity can be assessed by comparing the distribution of potential effect modifiers among the direct comparisons available in the network [13]. Information regarding patient and study characteristics that may act as effect modifiers were collected and are presented in Table 1.

### 2.10. Statistical Analysis

Direct estimates were obtained using a comparison-specific random-effects model. Subsequently, a random-effects network meta-analysis was conducted to simultaneously compare the relative effectiveness of all interventions [17]. A common heterogeneity (τ) was assumed across all comparisons and compared with previously derived empirical distributions for heterogeneity [18]. Heterogeneity was assessed with Cochran’s Q test and the I-squared (I^2^) statistic. The heterogeneity was classified as low (I^2^ < 25%), moderate (I^2^ < 50%), or substantial (I^2^ ≥ 75%). When heterogeneity was detected (I^2^ ≥ 50), a sensitivity analysis excluding the relevant studies was performed. For all possible pairwise comparisons, the odds ratio (OR) and 95% confidence interval (CI) was estimated using a multivariate meta-analysis approach, which treats different comparisons in studies as separate outcomes and accounts for correlation introduced by multi-arm trials [19]. The network meta-analysis models were conducted using a specific package (netmeta) in R. The analyses were performed on an intention-to-treat basis. To assess the ranking probabilities of each intervention, cumulative ranking curves were plotted, and the surface under these curves (surface under the cumulative ranking, SUCRA) was calculated. SUCRA represents a percentage and the effectiveness of an intervention compared to a theoretical intervention that is always assumed to be the best without uncertainty. A higher SUCRA value indicates a better rank for the intervention [20]. Contribution plots were created to evaluate the influence of each direct comparison on the network estimates and the overall network.

### 2.11. Assessment of Inconsistency

Consistency between direct and indirect estimates of treatment effects was examined to evaluate agreement within the network. An inconsistency plot was generated using the “netheat” command in R. Within each closed loop, an inconsistency factor (IF) was derived by comparing the odds ratios obtained from direct evidence with those inferred indirectly. Values approaching 1 indicate concordance between the two sources of evidence. If the 95% confidence interval does not include unity (1), significant inconsistency in a loop is detected. This analysis assumed a shared heterogeneity parameter across all comparisons, based on the estimate obtained from the network meta-analysis model.

### 2.12. Assessment of Small-Study Effects

To take into consideration the potential influence of small studies and publication bias, the effect was evaluated using a comparison-adjusted funnel plot. This plot considered the estimated effects of studies for different comparisons within the network.

## 3. Results

### 3.1. Search Results

**Figure 1:**
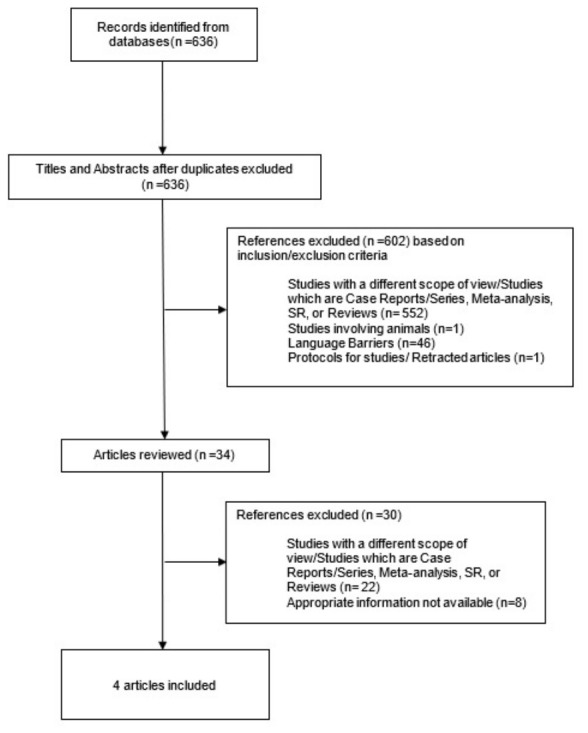
Inclusion process flowchart.

The initial electronic search initially identified 636 records. The selection process is illustrated in Figure 1. After 632 studies were excluded with their respective reasons, a total of four studies [10,14,15,16] published between 2002 and 2021 were included in the qualitative and quantitative synthesis involving 172 patients. The characteristics of these included studies are presented in Table 1.

### 3.2. Geometry of the Networks

**Figure 2:**
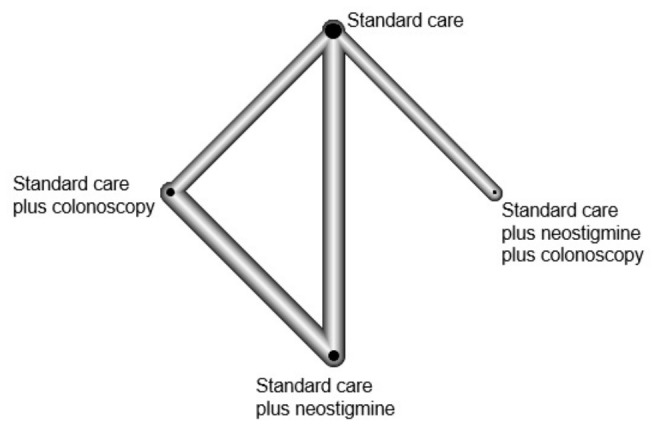
Network of the different interventions for the outcome of resolution of symptoms.

Figure 2 displays the network plot for the resolution of symptoms. In this analysis, all interventions were pairwise tested in four studies (12,28–30). Among these studies, the largest number of patients were included in the conservative supportive treatment, involving 70 patients across three studies [14,15,16]. Neostigmine administration on top of supportive treatment involved 44 patients across four studies (12,28,29). Colonoscopy on top of supportive treatment involved 40 patients across three studies (12,28,30). The combination of colonoscopy, neostigmine and supportive treatment involved 18 patients in one study (30).

### 3.3. Risk of Bias

The risk of bias of the included studies was assessed using the Newcastle–Ottawa Scale (NOS), and the results are summarized in Table 2. Overall, the methodological quality of the included studies was high. Three studies (28–30) were judged to be at low risk of bias, achieving high scores across all NOS domains. These studies demonstrated good representativeness of the exposed cohorts, appropriate selection of concurrent control groups, secure ascertainment of exposure, and complete follow-up. All studies adequately demonstrated that outcomes of interest were not present at baseline and controlled for the most important confounding factors. One study was judged to be at moderate risk of bias, primarily due to limited reporting on the ascertainment of outcomes, while performance across the remaining domains was comparable to that of the low-risk studies [14]. No study was classified as being at high risk of bias.

### 3.4. Assessment of Transitivity and Inconsistency

Among studies that compared more than one intervention (Table 1), no discrepancies were found in terms of study and participant characteristics, as well as the definition of intervention and outcomes. In order to assess transitivity, inconsistency was evaluated, but no evidence of inconsistency was observed.

### 3.5. Primary Outcome

For symptom resolution, the comparison between combined standard care and neostigmine versus standard care was informed by two studies, yielding a network odds ratio (OR) of 13.86 (95% CI 3.06–62.83). The corresponding direct and indirect estimates were 13.46 (95% CI 2.73–66.27) and 18.04 (95% CI 0.16–2079.58), respectively. The ratio of odds ratios (RoR) was 0.75 (95% CI 0.00–111.61; *p* = 0.91) (Table 3).

For the comparison between combined standard care and colonoscopy versus standard care, one study contributed to the analysis, yielding a network estimate odds ratio (OR) of 65.65 (95% CI 11.70–368.50), with direct and indirect estimates of 53.43 (95% CI 6.85–416.97) and 107.39 (95% CI 4.48–2571.61), respectively. The ratio of odds ratios (RoR) was 0.50 (95% CI 0.01–21.86; *p* = 0.72) (Table 3).

The comparison between combined standard care and colonoscopy and neostigmine versus standard care was informed by one study, having only a direct contribution of estimate of 4.27 (95% CI 0.60–30.35).

In the comparison of colonoscopy versus neostigmine, two studies contributed, resulting in a network OR of 4.74 (95% CI 1.17–19.25), with direct and indirect estimates of 5.49 (95% CI 1.33–22.64) and 0.00 (95% CI 0.00–66.93), respectively. The RoR was 1697.14 (95% CI 0.07–38,817,960.60; *p* = 0.15) (Table 3).

Comparisons between colonoscopy and combined colonoscopy with neostigmine and between neostigmine and combined colonoscopy with neostigmine therapy were informed exclusively by indirect evidence, yielding network ORs of 15.39 (95% CI 1.13–209.78) and 3.25 (95% CI 0.27–38.67), respectively (Table 3).

Regarding the SUCRA values, the combination of colonoscopy and standard care had the highest SUCRA value (98.9), followed by the combination of neostigmine and standard care (62.3), the combination of colonoscopy, neostigmine and standard care (38.4) and the last one is the standard care (9.8) (Figure S1).

### 3.6. Small-Study Effects

The comparison-adjusted funnel plots demonstrated symmetric distribution for all outcomes, indicating the absence of a significant small-study effect.

## 4. Discussion

### 4.1. Main Findings

In this network meta-analysis, we compared conservative supportive care, neostigmine, colonoscopic decompression, and their combinations for the resolution of symptoms in patients with Ogilvie’s syndrome. The analysis demonstrated that active interventions were associated with substantially higher odds of symptom resolution compared with standard supportive care alone. Among the evaluated strategies, the combination of colonoscopic decompression with supportive care ranked highest according to SUCRA values, followed by neostigmine combined with supportive care. Comparisons informed by both direct and indirect evidence showed no statistically significant inconsistency, supporting the internal coherence of the network. Although confidence intervals were wide for several estimates, reflecting sparse data, the relative ordering of interventions was consistent across analyses.

### 4.2. Comparison with Previous Studies

Previous systematic reviews have largely evaluated neostigmine and colonoscopic decompression separately, without formally integrating evidence across all available treatment strategies [21,22,23]. A recent systematic review by Zia et al. focused primarily on the efficacy of colonoscopic decompression and concluded that it may be superior to neostigmine, although relapse rates remained high and the evidence base was limited by heterogeneous study designs and small sample sizes [21]. Other narrative and pairwise meta-analyses have reported high initial success rates for neostigmine, typically exceeding 80%, but have emphasized the need for cardiac monitoring and the risk of recurrence [23,24].

Our findings extend the existing literature by directly comparing all commonly used management strategies within a single analytical framework. By leveraging both direct and indirect evidence, this network meta-analysis provides comparative estimates that are not available from traditional pairwise approaches. Importantly, our results suggest that combined strategies incorporating either neostigmine or colonoscopy alongside supportive care are associated with higher odds of symptom resolution than supportive care alone, supporting current stepwise management recommendations.

In comparison with previous studies, our analysis suggests that earlier escalation to active interventions (particularly in patients with significant colonic dilatation or failure to respond within 24–48 h of conservative management) potentially improves outcomes and reduces the risk of complications. Despite the fact that previous studies have positioned pharmacologic and endoscopic therapies as sequential alternatives, our findings highlight the potential benefit of an individualized, patient-centred approach. A combined or timely escalated approach should be considered according to clinical severity, comorbidity burden, and contraindications. Collectively, these results support refinement of current management algorithms by emphasizing early risk stratification and tailored intervention selection to optimize patient outcomes.

### 4.3. Interpretation

The observed differences in treatment effectiveness likely reflect the underlying pathophysiology of Ogilvie’s syndrome, in which autonomic imbalance leads to functional colonic obstruction [25,26]. Supportive care addresses reversible precipitating factors but may be insufficient once significant colonic dilation has developed. Neostigmine, by enhancing parasympathetic activity, directly targets impaired colonic motility, whereas colonoscopic decompression provides immediate mechanical relief of distension [27,28]. The higher ranking of combined approaches suggests that multimodal strategies may be particularly effective in patients who do not respond promptly to conservative measures alone.

The absence of statistically significant inconsistency between direct and indirect estimates strengthens confidence in the network estimates. However, the imprecision observed in several comparisons, particularly those informed by a limited number of studies, indicates that the magnitude of effect should be interpreted cautiously. The very large odds ratios observed in some indirect comparisons likely reflect sparse data rather than true differences in treatment efficacy.

### 4.4. Limitations

Several limitations should be acknowledged. First, the evidence base was dominated by observational studies, with only a small number of randomized or comparative studies contributing to the network. Although the overall risk of bias was judged to be low to moderate, residual confounding cannot be excluded. Second, sample sizes were small for several treatment nodes, resulting in wide confidence intervals and limited precision of effect estimates. Third, the definition of symptom resolution varied across studies, potentially introducing heterogeneity that could not be fully accounted for in the analysis. Fourth, data on adverse events associated with the included interventions, recurrence, and long-term outcomes were insufficient and inconsistently and inadequately reported across primary studies to allow meaningful comparative assessment beyond the primary outcome.

Finally, although no evidence of small-study effects or inconsistency was detected, the power to detect such phenomena was limited by the size and structure of the network.

### 4.5. Implications for Future Research

Future studies should prioritize well-designed randomized or pragmatic comparative trials evaluating neostigmine, colonoscopic decompression, and their combination using standardized outcome definitions. Larger multicenter cohorts would improve precision and allow exploration of clinically relevant subgroups, such as patients with severe colonic dilation or significant comorbidity. Incorporation of recurrence, complications, and patient-centred outcomes would further strengthen the evidence base and better inform clinical decision-making.

## 5. Conclusions

The findings indicate that active interventions, particularly colonoscopic decompression or neostigmine when used in conjunction with supportive care, are associated with higher odds of symptom resolution compared with supportive care alone. Among the evaluated approaches, colonoscopic decompression combined with supportive care ranked highest in effectiveness.

## Figures and Tables

**Table 1:** Characteristics of the included studies.

Study	Age	Study Population	Ethnicity	Intervention Groups
Liu et al. [14].	Mean: 67 Range: 20–93	80	White: 63% African American: 24%	Neostigmine Colonoscopic Decompression Standard Care
Williamson et al. [10].	Median: 68 IQR: 62–84	34	Not Applicable	Neostigmine Colonoscopic Decompression
Ponec et al. [15].	Median.s: 67 IQR: 40–82 Median.c: 64 IQR: 43–83	37	Not Applicable	Neostigmine Standard Care
Magda et al. [16].	Mean: 67 SD: 15	21	White: 59% Black: 38% Other: 3%	Neostigmine + Colonoscopic Decompression Standard Care

**Table 2:** Risk of bias assessment of included studies in network meta-analysis according to NOS. Each item is categorized as follows: † truly representative of average patients with Oglivie’s Syndrome; somewhat representative of Oglivie’s Syndrome; selected group; no description of derivation of cohort. ‡ Drawn from the same source as intervention cohort (concurrent controls); drawn from a different source; no description of derivation of exposed cohort. § Secure record; structured interview; written self-report; no description. ≠ Demonstration that outcome of interest was not present at the start of the study: yes or no. ¶ Study controls for the most important factor; study controls for any additional factor; not carried out or not reported. ȣ Independent blind assessment; record linkage; self-report; no description. || Follow-up enough for outcomes to occur: yes or no. ** Complete follow-up, all subjects are accounted for; subjects lost to follow-up were unlikely to introduce bias because small number were lost, >90% had follow-up or description was provided of those lost; follow-up rate <90% and no description of those lost; no statement.

Study	Representativeness of Exposure Cohort †	Selection of Non-Exposed Cohort ‡	Ascertainment of Exposure §	Incidence of Disease ≠	Comparability ¶	Ascertainment of Outcome ȣ	Length of Follow-Up ||	Adequacy of Follow-Up **	Overall
Liu et al. 2021 [14]	Somewhat representative	Concurrent controls	Secure record	Yes	Controls for the most important factor	Record linkage	Yes	Complete follow-up	Low risk
Williamson et al. 2023 [10]	Truly representative	Concurrent controls	Secure record	Yes	Controls for the most important factor	No precise description	Yes	Complete follow-up	Moderate risk
Magda et al. 2017 [16]	Truly representative	Concurrent controls	Secure record	Yes	Controls for the most important factor	Record linkage	Yes	Complete follow-up	Low risk
Ponec et al. 1999 [15]	Truly representative	Concurrent controls	Secure record	Yes	Controls for the most important factor	Independent blind assessment	Yes	Complete follow-up	Low risk

**Table 3:** Odds ratios and confidence intervals in direct and indirect contribution in grey. Odds ratios and confidence intervals in direct contribution only in white.

Standard Care	13.5 (2.7–66.3)	53.4 (6.8–416.7)	4.3 (0.6–30.3)
13.8 (3.06–62.8)	Neostigmine	5.5 (1.3–22.6)	
65.6 (11.7–368.5)	4.7 (1.2–19.2)	Colonoscopy	
4.3 (0.60–30.3)	3.2 (0.3–38.7)	15.4 (1.1–209.1)	Colonoscopy and Neostigmine

## Data Availability

No new data were created or analyzed in this study.

## Supplementary Materials

The following supporting information can be downloaded at: https://www.mdpi.com/article/10.3390/jcm15083177/s1, Figure S1: Rankogram of the different interventions. Table S1: PRISMA 2020 checklist [29].

## Abbreviations

The following abbreviations are used in this manuscript:

ACPO	Acute Colonic Pseudo-Obstruction
CI	Confidence Interval
OR	Odds Ratio
RoR	Ratio of Odds Ratios
