# Supplementary figures and images for: Management of Ogilvie’s Syndrome: A Network Meta-Analysis
Source: J Clin Med. 2026 Apr 21;15(8):3177. doi: 10.3390/jcm15083177 (PMC13117274; doi:10.3390/jcm15083177)

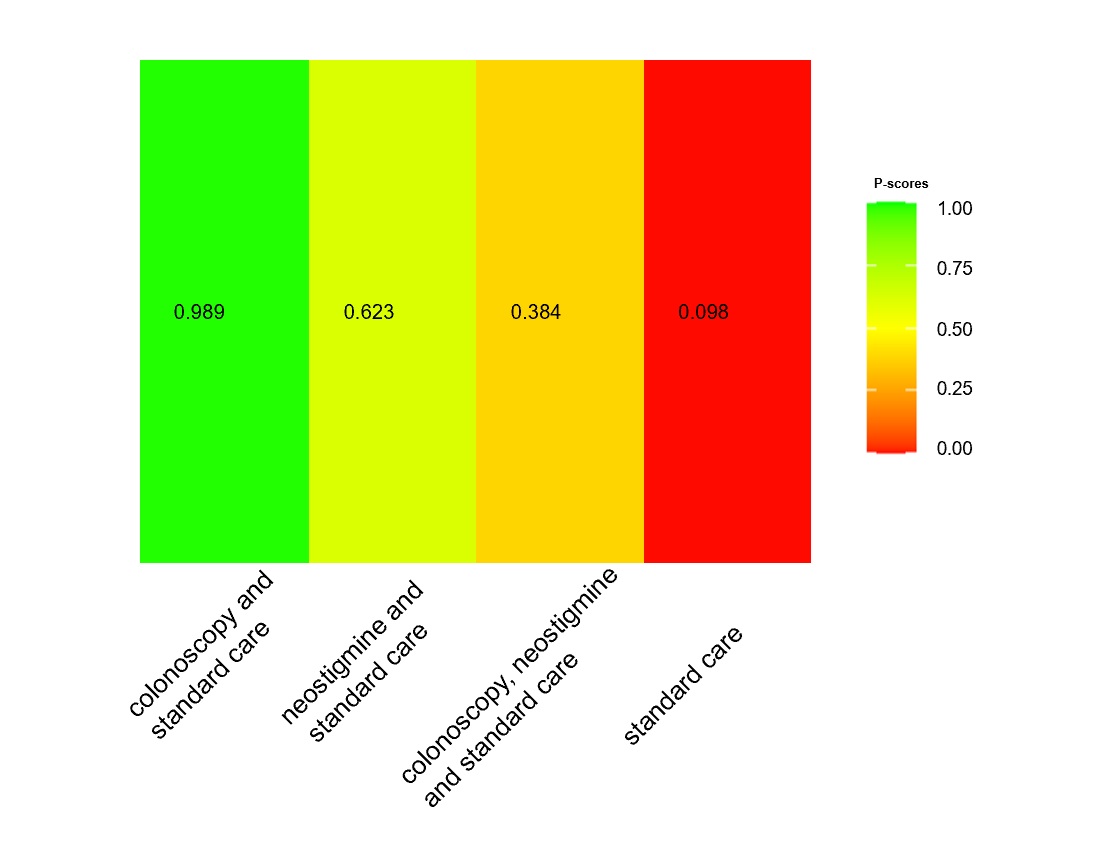

Supplement: Supplementary file 1 [file jcm-15-03177-s001.zip › Figure S1.jpg]
